# Distribution of selected carcinogenic hydrocarbon and heavy metals in an oil-polluted agriculture zone

**DOI:** 10.1007/s10661-014-4037-6

**Published:** 2014-10-02

**Authors:** E. O. Nwaichi, M. O. Wegwu, U. L. Nwosu

**Affiliations:** 1 Institute of Agrophysics, Lublin, Poland; 2 Department of Biochemistry, Faculty of Chemical Sciences, University of Port Harcourt, P.M.B. 5323, Port Harcourt, Rivers State Nigeria

**Keywords:** Carcinogenic hydrocarbons, Plants contaminant uptake and exposure, Oil pollution, Niger delta, Food chain

## Abstract

Owing to the importance of clean and fertile agricultural soil for the continued existence of man, this study investigated the concentrations of total petroleum hydrocarbons (TPHs), polycyclic aromatic hydrocarbons (PAHs) and some heavy metals in soils and selected commonly consumed vegetables and tubers from oil-polluted active agricultural farmland in Gokana of Ogoniland, Rivers State, Nigeria. Samples from Umuchichi, Osisioma Local Government Area in Abia State, Nigeria, a non-oil-polluted area constituted the control. In test and control, up to 3,830 ± 19.6 mgkg^−1^ dw and 6,950 ± 68.3 mgkg^−1^ dw (exceeding DPR set limits) and 11.3 ± 0.04 mgkg^−1^ dw and 186 ± 0.02 mgkg^−1^ dw for TPH and PAHs, respectively, were recorded in test soil and plant samples, respectively. Among the metals studied (Pb, Cd, Cr, Mn, Fe and Zn), Pb and Cr uptake exceeded WHO set limits for crops in test samples. Combined sources of pollution were evident from our studies. Bitterleaf and Waterleaf could be tried as bioindicators owing to expressed contaminants uptake pattern.

## Introduction

Nigeria is a major producer and exporter of crude petroleum oil and also an important agricultural nation in the West African sub-region (Agbogidi et al. 2005). As crude oil comes from the well, it contains mixture of hydrocarbon compounds and relatively small quantities of other materials such as oxygen, nitrogen, sulphur, salt, water and some trace metals. In the refinery, most of these non-hydrocarbon substances are removed and the oil is broken down into useful products (Nwaichi et al. 2011). The soil is very important to human existence for various reasons, especially for agriculture and has been subjected to various abuses including spillage of petroleum (crude oil) and petroleum-by products, dumping of waste and other contaminating activities (Osam 2011). Soil contamination has been a growing concern since it can be a source of groundwater (drinking water) contamination and can also reduce the usability of land for development. Elevated levels of some heavy metals in different parts of the globe have increased the interest for environmentalists and ecotoxicologists in toxicity and environmental degradation. Humans and ecosystem may be exposed to chemical hazards such as heavy metals through direct ingestion of contaminated soil, consumption of crops and vegetables grown on the contaminated lands or drinking water that has percolated through such soils (Mclaughlin et al. 2000). These pollutants may cause long- or short-term damage by changing the growth rate of plant or animal species, or by interfering with human amenities, comfort, health or property values (Tietenberg 2006). The definition of crude oil and gas pollution in this study embraces oil spillages on crop farms, areas of crop farms occupied by flow stations, oil wells, gas flaring sites, pipeline laying sites, borrow pits and other oil exploration, exploitation and related activities. Within the European community, 11 elements of highest concern are arsenic, cadmium, cobalt, chromium, copper, mercury, manganese, nickel, lead, tin and thallium (MEPPRM 2014); the emissions of which are regulated in waste incinerators. Some of these elements are actually necessary for humans in minute amounts (cobalt, copper, chromium, manganese, nickel) while others are carcinogenic or toxic, affecting, among others, the central nervous system (manganese, mercury, lead, arsenic), the kidneys or liver (mercury, lead, cadmium, copper) or skin, bones or teeth (nickel, cadmium, copper, chromium) (Zevenhoven and Kilpinen 2001). Cadmium, lead and zinc are also released in tiny particulates as dust from rubber tyres on busy road surfaces; the small size allows these toxic metals to rise in the wind to be inhaled, or transported onto topsoil or edible plants through precipitation of their compounds or by ion exchange into soils and muds. Heavy metal pollutants can localize and lay dormant, and this can have multiple effects on the environment.

Polycyclic aromatic hydrocarbons (PAHs) are produced from incomplete combustion of organic materials, fossil fuels, petroleum product spillage and various domestic and industrial activities (Johnsen et al. 2005). Based on their ecotoxicity, the United States Environmental Protection Agency has prioritized 16 PAHs as environmental pollutants (Nwaichi et al. 2010). Total petroleum hydrocarbons (TPH) are the measurable amount of petroleum-based hydrocarbons in an environmental media (Rauckyte et al. 2010).

Oil exploration in Ogoniland commenced in the 1950s and extensive production facilities were established. Ogoniland is situated in an area 1,000 km^2^ east of Port Harcourt in Rivers State, Nigeria. The area has a tragic history of pollution from oil spills; oil well fires, environmental incidents, such as spills and uncontrolled flares (UNEP, United Nations Environment Programme 2011). Gokana is one of the six kingdoms of Ogoniland. It has a rain forest and most dwellers are famous farmers. It lies on the coastal lowland in the south eastern part of Rivers State and is characterised by high rainfall (2,000–2,500 mm/yr), high temperature and high humidity. Gokana is located between latitude 4°36 N and longitude 7°21 E of the equator. The control area, Umuchichi village in Osisioma LGA, is a coastal plain located on the southern part of Abia State, Nigeria and lies 4°40 and 6°14 N and 7°10 and 8° E. It is a non-polluted area with less industry presence.

This study therefore seeks to evaluate the distribution of some heavy metals, TPH and PAHs in selected regularly-consumed food crops and soils from an oil-polluted active agricultural farmland and making comparisons with a view to health implications.

## Materials and methods

### Sample sourcing

Soil Samples (in random replicates of three) were taken from oil-polluted active agricultural farmlands in Gokana (test) and non-oil-polluted active agricultural farmlands in Umuchichi (control). Leafy vegetables (Bitterleaf or *Vernonia amygdelina* and Waterleaf or *Talinum triangulare*) and tuber crops (Cassava or *Manihot esculenta* and Cocoyam or *Xanthotosoma sagittifolium*) were freshly harvested from these locations and were collected following standard environmental sampling protocols (US. EPA 1986).

### Sample preparation and analysis

Soil samples were air dried, crushed and sieved (2 mm screen). For heavy metals, 5 g of each sample was weighed into a clean, dry silica dish, covered and ignited in a furnace for 6 h at 500 °C until a grey white ash was obtained. The cover of the dish was opened to allow for escape of gases. To cool ash samples, 5 ml of 10 % HCl was added to enhance dissolution and 5 ml of 10 % HNO_3_ was added thereafter and set on a water bath to dissolve completely. The solution was transferred into a clean dry 50 ml standard volumetric flask and marked up with distilled water (Khan et al. 2008). The concentrations of Fe, Mn, Zn, Cu, Cd and Pb in the filtrate were determined by atomic absorption spectrometry (ContrAA 300, Analytik Jena, Germany). The blank reagent and standard reference soil materials were included in each sample batch to verify the accuracy and precision of the digestion procedure and also for subsequent analyses. For TPHs and PAHs, 1 g sample was weighed into a clean extraction container and 10 ml dichloromethane (extraction solvent) was added. This was allowed to settle after thorough mixing. The mixture was carefully filtered into extraction bottle using clean filter paper fitted in a Buchner funnel, and the extract was concentrated to 2 ml and then transferred for separation in a HP gas chromatograph 5890 series II. About 8–10 ml of the eluent/extract was collected and labelled aromatics (API, American Petroleum Institute 1994). Using a hypodermic syringe, 1 μL of the concentrated aromatic fraction was injected through a rubber septum into the column. Separation occurred as the vapour constituent’s partition between the gas and liquid phases and detection was possible with FID. On the other hand, samples of various vegetables and tubers were washed with distilled water to remove loose particles. Vegetable samples and chopped tubers were sun dried for 4 days and ground in a high speed plastic blender (SON Binatone) for several minutes until they became homogenous. Similar protocol as described earlier was followed for hydrocarbons analyses. Data were validated by reviewing for completeness, holding times, calibrations (initial and continuing), specific blank analysis, GC tuning and system performance, surrogate recoveries, field replication precision, compound quantitation and detection limits.

### Statistical analysis

Obtained data were subjected to one-way analysis of variance (ANOVA) using STATISTICA vs 10, and test of significance was done at 95 % confidence level.

## Results and discussion

Quality control criteria were acceptable following the laboratory results analysed for the PAHs. Data were analysed to determine if any statistically marked differences existed between the datasets for the soils and plant tissue types. While impacted vegetation presented with brownish to yellowish coloration and stunted growth, those located 50 m away from the impacted site appeared greenish and healthy. From Tables 1 and 2, average individual concentrations of TPHs ranged from below detection limit of 0.0001–0.58 ± 0.001 mgkg^−1^ dw for control samples, with highest concentration recorded for C-21 in *Talinum triangulare* which differed marginally from 0.11 to 0.00 for C-31 in *Vernonia amygdelina*. Cumulative TPHs distribution, however, gave the pattern *V. amygdelina* > *T. triangulare* > *X. sagittifolium* > *M. esculenta* > soil (Table 1), as all soil values fell below the detection limit of the analytical instrument. According to DEQ (2000), gasoline and condensate range organics (GRO) generally include C4 through C9 hydrocarbons, diesel range organics (DRO) generally include C10 through C24, while crude oil, in general, includes C5 through C34 hydrocarbons. It is therefore presumed that meagre concentrations observed in control tissue samples could be attributed to neighbouring gas flaring activities. For the test samples, however, observed data ranged from below detection limit to 2,280 ± 2.89 mgkg^−1^ dw and this highest fractional mean TPH concentration was recorded in soil for C-14 components. As indicated in Table 1, GC analysis revealed the presence of pristane (C_19_H_40_) and phytane (C_20_H_42_) in all the polluted samples (Table 2). These two acyclic isoprenoid hydrocarbons serve as biomarkers in spilled oil. The disappearance of the lighter molecular weight hydrocarbon numbers below C12 in most of the test samples may indicate that the oil was slightly weathered after spill incident and hence, greater susceptibility to secondary processes of evaporative weathering and biodegradation. The plant samples similarly accumulated TPHs in the order: *V. amygdelina* > *T. triangulare* > *X. sagittifolium* > *M. Esculenta*, but were markedly (*P* ≤ 0.05) low compared to soil total concentrations. It is noteworthy to mention that recorded cumulative TPHs levels (3,830 ± 19.6 mgkg^−1^ dw) in test soil far exceeded the maximum permissible limit of 50 mgkg^−1^ dw by Department of Petroleum Resources (DPR). Total mean soil levels in control samples were significantly lower (with values: <0.0001, 0.58 ± 0.04, 0.49 ± 0.02, 0.14 ± 0.01 and 0.05 ± 0.004 mgkg^−1^ dw) than those of test (with values: 3824.12 ± 19.6, 11.34 ± 0.07, 9.67 ± 0.08, 3.99 ± 0.09 and 2.17 ± 0.06 mgkg^−1^ dw) for all samples. Up to 35.2 mgkg^−1^ dw phythane was observed for test soil in contrast to non-detectable levels in control. Secondary evaluation (Table 3) for such soil, with multiple contaminants gave value for the sum of the individual Ri values (ΣRi) >1. This implies further evaluation, potentially through site-specific risk assessment.

**Table 1:** A mean TPH (mgkg^−1^) levels in control area (Umuchichi)

Components	Soil	Bitterleaf	Waterleaf	Cocoyam	Cassava
C-8	<0.001	<0.001	<0.001	<0.001	<0.001
C-9	<0.001	<0.001	<0.001	<0.001	<0.001
C-10	<0.001	<0.001	<0.001	<0.001	<0.001
C-11	<0.001	<0.001	<0.001	<0.001	<0.001
C-12	<0.001	<0.001	<0.001	<0.001	<0.001
C-13	<0.001	<0.001	<0.001	<0.001	<0.001
C-14	<0.001	0.03 ± 0.00^b^	<0.001	<0.001	<0.001
C-15	<0.001	0.01 ± 0.00^a^	<0.001	<0.001	<0.001
C-16	<0.001	0.01 ± 0.00^a^	<0.001	<0.001	<0.001
C-17	<0.001	0.01 ± 0.00^a^	<0.001	<0.001	<0.001
Pristane	<0.001	0.05 ± 0.00^d^	<0.001	<0.001	<0.001
C-18	<0.001	0.01 ± 0.00^a^	<0.001	<0.001	<0.001
Phythane	<0.001	0.02 ± 0.00^a^	<0.001	<0.001	<0.001
C-19	<0.001	0.02 ± 0.00^a^	<0.001	<0.001	<0.001
C-20	<0.001	0.02 ± 0.00^d^	0.01 ± 0.00^e^	0.01 $\pm 0.00$ ^a^	<0.001
C-21	<0.001	0.02 ± 0.00^a^	0.12 ± 0.00^a^	<0.001	<0.001
C-22	<0.001	0.01 ± 0.00^a^	0.01 ± 0.00^a^	<0.001	<0.001
C-23	<0.001	0.01 ± 0.00^a^	<0.001	<0.001	<0.001
Components	Soil	Bitterleaf	Waterleaf	Cocoyam	Cassava
C-24	<0.001	0.01 ± 0.00^a^	<0.001	<0.001	<0.001
C-25	<0.001	0.01 ± 0.00^a^	<0.001	<0.001	<0.001
C-26	<0.001	0.02 ± 0.00^b^	<0.001	<0.001	<0.001
C-27	<0.001	0.01 ± 0.00^d^	0.01 ± 0.00^d^	<0.001	<0.001
C-28	<0.001	0.02 ± 0.00^d^	0.02 ± 0.00^d^	<0.001	<0.001
C-29	<0.001	0.03 ± 0.00^e^	0.01 ± 0.00^a^	<0.001	<0.001
C-30	<0.001	0.02 ± 0.00^a^	0.01 ± 0.00^a^	<0.001	<0.001
C-31	<0.001	0.11 ± 0.00a	0.01 ± 0.00^a^	0.01 ± 0.00^a^	<0.001
C-32	<0.001	0.01 ± 0.00^e^	0.03 ± 0.00^d^	0.02 ± 0.00^a^	<0.001
C-33	<0.001	0.04 ± 0.00^c^	0.09 ± 0.00^a^	0.01 ± 0.00^d^	<0.001
C-34	<0.001	0.03 ± 0.00^d^	0.05 ± 0.00^e^	0.05 ± 0.00^c^	0.01 ± 0.00^a^
C-35	<0.001	0.03 ± 0.00^c^	0.03 ± 0.00^e^	0.01 ± 0.00^d^	<0.001
C-36	<0.001	0.02 ± 0.00^c^	0.03 ± 0.00^a^	0.01 ± 0.00^d^	0.01 ± 0.00^b^
C-37	<0.001	0.01 ± 0.00^c^	0.02 ± 0.00^b^	0.01 ± 0.00^a^	<0.001
C-38	<0.001	0.01 ± 0.00^b^	<0.001	<0.001	<0.001
C-39	<0.001	<0.001	0.03 ± 0.00^a^	<0.001	<0.001
C-40	<0.001	0.02 ± 0.00^a^	<0.001	<0.001	<0.001
Total	<0.001	0.58 ± 0.04^b^	0.49 ± 0.02^b^	0.14 ± 0.01^c^	0.05 ± 0.00^d^

Values with different superscript letters (a, b, c, d, e) in the same row are significantly different at 0.05 level (*P* ≤ 0.05)

**Table 2:** A mean level of TPH (mgkg^−1^) in test (Gokana) area

Components	Soil	Bitterleaf	Waterleaf	Cocoyam	Cassava
C-8	<0.001	<0.001	<0.001	<0.001	<0.001
C-9	<0.001	<0.001	0.01 ± 0.00^a^	<0.001	<0.001
C-10	<0.001	0.01 ± 0.00^b^	0.01 ± 0.00^a^	<0.001	<0.001
C-11	<0.001	0.01 ± 0.00^b^	0.02 ± 0.00^a^	<0.001	<0.001
C-12	<0.001	0.02 ± 0.00^a^	0.01 ± 0.00^b^	<0.001	<0.001
C-13	224 ± 7.77^a^	0.53 ± 0.00^b^	0.23 ± 0.00^b^	0.02 ± 0.00^b^	0.01 ± 0.00^b^
C-14	2,280 ± 2.89^a^	0.35 ± 0.00^b^	0.07 ± 0.00^b^	0.14 ± 0.00^b^	0.07 ± 0.00^b^
C-15	47.1 ± 26.5^c^	4.00 ± 0.06^b^	2.73 ± 0.06^a^	0.31 ± 0.00^d^	0.43 ± 0.00^d^
C-16	60.2 ± 0.06^a^	0.10 ± 0.00^c^	0.20 ± 0.00^b^	0.09 ± 0.00^c^	0.08 ± 0.00^c^
C-17	289 ± 1.53^a^	<0.001	0.01 ± 0.00^b^	0.03 ± 0.00^b^	0.01 ± 0.01^b^
Pristane	<0.001	0.22 ± 0.01^d^	0.21 ± 0.00^d^	0.04 ± 0.00^c^	0.05 ± 0.02^a^
C-18	189 ± 1.15^a^	0.03 ± 0.00^b^	0.07 ± 0.00^b^	0.22 ± 0.00^b^	0.03 ± 0.00^b^
Phythane	35.2 ± 1.34^a^	0.20 ± 0.00^b^	<0.001	0.02 ± 0.00^b^	0.04 ± 0.00^b^
C-19	20.8 ± 0.12^d^	1.23 ± 0.01^c^	0.31 ± 0.00^a^	0.03 ± 0.00^b^	0.03 ± 0.00^b^
C-20	297 ± 1.15^a^	0.11 ± 0.00^b^	0.11 ± 0.00^b^	0.05 ± 0.00^b^	0.17 ± 0.00^b^
C-21	26.9 ± 0.23^a^	0.17 ± 0.00^b^	0.18 ± 0.00^b^	0.04 ± 0.00^b^	0.03 ± 0.00^b^
C-22	32.3 ± 0.10^a^	1.39 ± 0.09^b^	0.15 ± 0.00^c^	0.06 ± 0.00^c^	0.02 ± 0.00^b^
C-23	219 ± 2.31^a^	0.01 ± 0.00^b^	1.90 ± 0.02^b^	2.12 ± 0.01^b^	0.02 ± 0.00^b^
Total	3720.5	8.38	6.22	3.17	0.99
Components	Soil	Bitterleaf	Waterleaf	Cocoyam	Cassava
C-24	9.55 ± 0.01^d^	1.36 ± 0.04^c^	0.24 ± 0.00^a^	0.12 ± 0.00^b^	0.16 ± 0.00^b^
C-25	1.25 ± 0.01^e^	0.09 ± 0.00^b^	0.21 ± 0.00^c^	0.18 ± 0.02^a^	0.30 ± 0.00^d^
C-26	11.7 ± 0.15^c^	0.30 ± 0.00^d^	0.20 ± 0.00^d^	0.04 ± 0.00^a^	0.03 ± 0.00^b^
C-27	6.31 ± 0.01^a^	0.05 ± 0.02^b^	0.04 ± 0.01^b^	0.04 ± 0.02^b^	0.30 ± 0.01^b^
C-28	5.33 ± 0.01^d^	0.08 ± 0.00^b^	0.16 ± 0.00^b^	0.04 ± 0.00^c^	0.07 ± 0.00^a^
C-29	1.28 ± 0.02^e^	0.03 ± 0.00^b^	0.01 ± 0.00^c^	<0.001	<0.001
C-30	11.0 ± 0.12^a^	0.03 ± 0.00^b^	0.01 ± 0.00^b^	0.03 ± 0.00^b^	0.03 ± 0.00^b^
C-31	18.8 ± 0.35^a^	<0.001	0.06 ± 0.00^b^	<0.001	0.03 ± 0.00^b^
C-32	2.54 ± 0.01^c^	<0.001	0.02 ± 0.00^a^	0.02 ± 0.00^b^	0.01 ± 0.00^d^
C-33	1.81 ± 0.02^c^	<0.001	0.02± 0.00^b^	0.06 ± 0.00^d^	0.05 ± 0.00^a^
C-34	8.91 ± 0.03^a^	0.01 ± 0.00^b^	0.67 ± 0.00^c^	0.01 ± 0.00^b^	0.05 ± 0.00^b^
C-35	6.91 ± 0.01^e^	0.66 ± 0.00^d^	0.51 ± 0.00^c^	0.02 ± 0.00^b^	0.06 ± 0.00^a^
C-36	2.03 ± 0.01^a^	<0.001	<0.001	<0.001	0.06 ± 0.00^b^
C-37	4.46 ± 0.01^a^	0.08 ± 0.00^b^	0.01 ± 0.00^c^	<0.001	0.02 ± 0.00^b^
C-38	4.49 ± 0.01^d^	0.04 ± 0.00^c^	0.05 ± 0.00^c^	0.01 ± 0.00^b^	0.03 ± 0.00^a^
C-39	1.66 ± 0.01^a^	0.12 ± 0.00^b^	0.02 ± 0.00^c^	<0.001	0.01 ± 0.00^c^
C-40	0.97 ± 0.01^b^	0.14 ± 0.00^a^	1.25 ± 0.01^b^	0.14 ± 0.02^b^	0.12 ± 0.00^b^
Total	3,830 ± 19.6^a^	11.3 ± 0.07^b^	9.67 ± 0.08^c^	3.99 ± 0.09^d^	2.17 ± 0.06^e^

**Table 3:** Evaluation of TPH (mgkg^−1^) contaminants in study soil

Source	Chemical contaminant	Soil CTVi	Ci		Ri(Ci/CTVi)		Further evaluation needed?(is Ri > 1)	
			Test	Control	Test	Control	Test	Control
Gasoline	TPH GROa	2.80E+01	383E+03	1.0E−03	136.76	3.57E−5	Yes	No
Diesel or crude oil	TPH DRO	2.30E+03	4.69E+03	1.0E−03	1.67	4.35E−7	Yes	No
ΣRi					138.43	3.61E−5	Yes	No

*a* is based on fate and transport evaluation for protection of groundwater. *Ri C/CTVi* where: *Ri* is the risk posed by contaminant “i”, *Ci* is the maximum concentration of contaminant “i” in soil, in mgkg^−1^ and *CTVi* is the soil cleanup level provided on the cleanup level look-up table, in mgkg^−1^ (MDNR and Michigan Department of Natural Resources 1993; DEQ 2000). For multiple contaminants in soil, if the sum of the individual Ri values (ΣRi) >1, further evaluation would be required, potentially through site-specific risk assessment

**Fig. 1:**
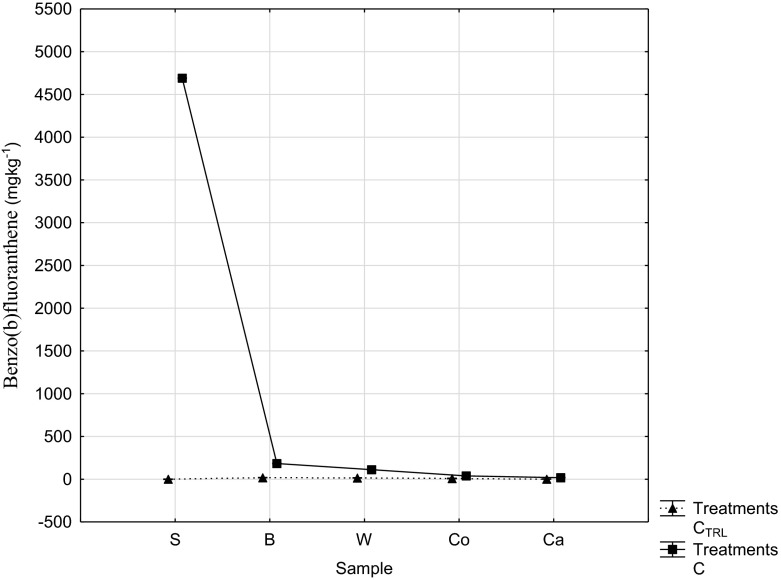
Comparative significance of carcinogenic bezo(**b**)fluoranthene distribution; *S* soil, *B* Bitterleaf, *W* Waterleaf, *Co* Cocoyam, *Ca* Cassava, *C* contaminated, *C*_*TRL*_ Control. *Vertical bars* denote 0.95 confidence intervals

Mean individual component concentrations of PAHs in all control samples (Table 4) ranged from below detection limit of <0.0001 to 18.4 ± 2.3 mgkg^−1^ dw and from <0.0001 to 4,690 ± 2.71 mgkg^−1^ dw for test samples. It is noteworthy that these upper limits were recorded for carcinogenic benzo (b) fluoranthene in both control and test and showed similar trend in plant tissues especially for Bitterleaf and Waterleaf (Fig. 1) and were statistically significant (at 0.95 confidence levels). This is attributable to several oil spills and other exploration-related activities in the area. Its persistence in the environment and poor degradability is probably due to its high molecular weight (Giridhar, P. & Krishna, P 2010). Their presence in crude oil makes differentiation of sources possible (Osuji and Onojake 2006) using useful ratios. All PAHs were below detection limit (<0.0001) in control soil but gave significant values (*P* < 0.05) in corresponding plant samples. Potential danger is inevitable as elevated levels of strongly carcinogenic benzo (a) pyrene exceeded DPR intervention limit of 0.01 mgkg^−1^ dw for food (Table 5) and intensely exceeded common regulatory target cleanup levels for PAHs and B(*a*)P-TE (0.1–0.66 mgkg^−1^) as reported by Bradley et al. (1994). He presented urban surface soil results (upper 95 % confidence interval of 3.3 and 12.4 mgkg^−1^ for total B(*a*)P-TE and total PAHs, respectively) that were much below the background concentrations of these compounds in test (Gokana) soils. The total mean concentrations of PAHs of 6,950 μgkg^−1^ in soil from study area (Gokana) exceeded the Department of Petroleum Resources permissible limit of 1 mgkg^−1^ (1,000 μgkg^−1^) (DPR 1991) and were significant in comparison to control. However, the levels in crop samples did not exceed the EU limit of 0.2 mgkg^−1^ (200 μgkg^−1^) (BFR 2010) for consumer products. No wonder, the natives complain of rashes, blisters, coughing, throat irritation, red, watery and itchy eyes and these signs and more, have been reported by Aguilera et al. (2010) from ingestion of hydrocarbons and dermal exposure. Owing to their low solubility and a high tendency to interact with non-aqueous phases, high molecular weight PAHs become potentially unavailable for degradation by microbes, which tend to favourably degrade compounds dissolved in water (Miller et al. 2004).

**Table 4:** Mean levels of PAHs (mgkg^−1^) in control area

Components	Soil	Bitterleaf	Waterleaf	Cocoyam	Cassava
Naphthalene	<0.001	<0.001	<0.001	<0.001	<0.001
Acenaphthylene	<0.001	<0.001	<0.001	<0.001	<0.001
Acenaphthene	<0.001	0.01 ± 0.01^a^	0.01 ± 0.01^a^	0.01 ± 0.01^a^	0.01 ± 0.01^a^
Fluorene	<0.001	<0.001	<0.001	<0.001	<0.001
Phenanthrene	<0.001	0.17 ± 0.04^a^	<0.001	<0.001	<0.001
Anthracene	<0.001	<0.001	0.29 ± 0.05^a^	<0.001	<0.001
Floranthene	<0.001	<0.001	<0.001	<0.001	<0.001
Pyrene	<0.001	<0.001	<0.001	0.03 ± 0.02^c^	<0.001
Benz(a)anthracene	<0.001	<0.001	<0.001	<0.001	<0.001
Chrysene	<0.001	<0.001	<0.001	<0.001	<0.001
Benzo(b)fluoranthene	<0.001	18.4 ± 2.31^b^	15.1 ± 1.77^a^	8.69 ± 2.42^c^	2.03 ± 0.03^b^
Benzo(k)fluoranthene	<0.001	<0.001	0.01 ± 0.01^a^	0.01 ± 0.01^c^	0.01 ± 0.01^c^
Benzo(a)pyrene	<0.001	<0.001	0.03 ± 0.02^a^	<0.001	<0.001
Indeno(1,2,3-cd)pyrene	<0.001	0.10 ± 0.00^c^	0.28 ± 0.03^d^	0.08 ± 0.00^c^	0.05 ± 0.00^a^
Dibenz(a,h)anthracene	<0.001	<0.001	0.01 ± 0.01^b^	<0.001	<0.001
Benzo(g,h,i)perylene	<0.001	<0.001	<0.001	<0.001	<0.001
Total	<0.001	18.6 ± 0.02^b^	15.8 ± 0.10^b^	8.83 ± 0.02^c^	2.11 ± 0.02^d^

**Table 5:** Mean levels of PAHs (mgkg^−1^) in test area

Components	Soil	Bitterleaf	Waterleaf	Cocoyam	Cassava
Naphthalene	<0.001	0.01 ± 0.00^a^	<0.001	<0.001	<0.001
Acenaphthylene	<0.001	0.03 ± 0.00^c^	0.06 ± 0.00^a^	0.02 ± 0.00^b^	0.01 ± 0.00^c^
Acenaphthene	297 ± 1.43^a^	0.34 ± 0.00^b^	<0.001	<0.001	<0.001
Fluorene	290 ± 1.51^a^	0.06 ± 0.00^b^	0.01 ± 0.00^c^	<0.001	<0.001
Phenanthrene	278 ± 1.78^a^	0.03 ± 0.00^b^	0.01 ± 0.00^c^	<0.001	<0.001
Anthracene	323 ± 2.89^a^	1.48 ± 0.04^b^	0.65 ± 0.00^b^	<0.001	<0.001
Floranthene	110 ± 2.67^a^	0.03 ± 0.01^b^	<0.001	<0.001	<0.001
Pyrene	57.1 ± 3.22^b^	0.09 ± 0.03^a^	<0.001	<0.001	<0.001
Benz(a)anthracene	102 ± 2.25^a^	0.01 ± 0.01^b^	<0.001	<0.001	<0.001
Chrysene	99.1 ± 6.21^a^	0.04 ± 0.00^b^	<0.001	<0.001	<0.001
Benzo(b)fluoranthene	4,690 ± 2.71^a^	183 ± 1.56^b^	112 ± 1.03^c^	37.8 ± 0.77^d^	18.0 ± 0.91^e^
Benzo(k)fluoranthene	47.0 ± 4.91^a^	0.17 ± 0.00^b^	0.04 ± 0.00^b^	<0.001	0.02 ± 0.00^d^
Benzo(a)pyrene	47.4 ± 3.89^a^	0.40 ± 0.05^b^	0.07 ± 0.00^c^	0.15 ± 0.00^b^	0.01 ± 0.00^d^
Indeno(1,2,3-cd)pyrene	694 ± 23.6^a^	0.13 ± 0.03^b^	0.75 ± 0.05^c^	0.39 ± 0.06^c^	0.61 ± 0.05^c^
Dibenz(a,h)anthracene	0.55 ± 0.13^a^	0.01 ± 0.00^b^	0.01 ± 0.00^b^	<0.001	<0.001
Benzo(g,h,i)perylene	4.14 ± 1.17^a^	0.01 ± 0.01^b^	<0.001	<0.001	<0.001
Total	6,950 ± 68.3^a^	186 ± 1.74^b^	114 ± 4.06^c^	18.7 ± 0.77^d^	18.7 ± 0.92^e^

Secondary evaluation of carcinogenic PAHs (Table 6) for such soil with multiple contaminants showed that the sum of the individual Ri values (ΣRi) >1, implying further evaluation, potentially through site-specific risk assessment. Heavy metal distribution in test and control samples indicated serious threats as observed Cr uptake levels for test and control and Pb in test only exceeded given EU (CEC 2006) and WHO limits for such soil (Tables 7 and 8). As would be expected, Mn and Zn (as micronutrients) levels in control were lower than those in test soils while abundant Fe levels were typical of soils in the Niger Delta. Toxic effects begin to occur at doses above 10–20 mgkg^−1^ of elemental iron, and ingestions of more than 50 mgkg^−1^ of elemental iron are associated with severe toxicity (Iron poisoning. http://www.webmd.com/first-aid/drug-overdose-poisoning-directory. Retrieved 2012). The first indication of iron poisoning by ingestion is a pain in the stomach, as the stomach lining becomes ulcerated. Tissue Pb levels for Bitterleaf and Cocoyam samples exceeded EU set limit of 0.3 mgkg^−1^ while Cr level exceeded WHO set limit of 0.05 mgkg^−1^ for Bitterleaf but fell below acute oral toxicity range of between 1,900 and 3,300 μgkg^−1^ risk (Monica et al. 2011). Discrepancies in Mn, Zn and Cr levels among the different samples analysed in control area may be attributed to accumulation of heavy metals from other sources including dump sites, use of fertilisers and aerial depositions. Bioaccumulation of metal contaminants may be a serious concern for Bitterleaf consumers. Measured Cd levels were below 0.1 mgkg^−1^ limit set by CODEX (2007). Heavy metals uptake in plants varied directly with soil concentrations and agreed with the findings of Lecoultre (2001) that uptake was higher in soil areas with higher concentrations. Elevated Cr levels in control samples may have arisen from flooding, which could mobilise heavy metals from soils particularly when readily oxidizable organic nutrients are available. This is possible as records of annual rainfall exceeded 2,000–2,500 mm/year in the area. Non-nutritive Pb gave marked (*P* ≤ 0.05) elevated levels (above WHO limits of 0.3 mgkg^−1^) in all test samples except for cassava samples. Lead interferes with a variety of body processes and is toxic to many organs and tissues including the heart, bones, intestines, kidneys, reproductive and nervous systems. It interferes with the development of the nervous system and is therefore particularly toxic to children, causing potentially permanent learning and behaviour disorders. Symptoms include abdominal pain, confusion, headache, anaemia, irritability and in severe cases, seizures, coma and death. A foetus developing in the womb of a woman who has elevated blood lead level is also susceptible to lead poisoning and is at greater risk of being born prematurely or with a low birth weight (Woolf et al. 2007). Children are more at risk for lead poisoning because their smaller bodies are in a continuous state of growth and development (Landrigan et al. 2002) and a threatening situation may result in study area in the nearest future. Many people, therefore, could be at risk of singly or combined adverse effects resulting from consumption of garden vegetables or tubers cultivated from oil-polluted soil and beyond, as supplies of these commodities are not limited.

**Table 6:** Evaluation of carcinogenic PAHs (mgkg^−1^) in study soils

Chemical contaminant	Soil CTVi	Ci		Ri(Ci/CTVi)		Further Evaluation needed?(is Ri > 1)	
		Test	Control	Test	Control	Test	Control
Benz(a)anthracene	1.50E−01	1.02E+02	1.0E−03	680	0.007	Yes	No
Benzo(b)fluoranthene	3.30E−01	4.69E+03	1.0E−03	14,212	0.003	Yes	No
Benzo(k)fluoranthene	3.30E−01	4.70E+01	1.0E−03	142.4	0.003	Yes	No
Dibenz(a,h)anthracene	3.30E−01	5.50E−01	1.0E−03	166.7	0.003	Yes	No
Indeno(1,2,3-cd)pyrene	3.30E−01	6.94E+02	1.0E−03	2,103	0.003	Yes	No
Chrysene	3.30E−01	9.91E+01	1.0E−03	300.3	0.003	Yes	No
Benzo(a)pyrene	1.50E−02	4.74E+01	1.0E−03	3,160	0.067	Yes	No
ΣRi				20,764	0.089	Yes	No

**Table 7:** Mean levels (mgkg^−1^) of studied metals in control area (Umuchichi)

	Soil	Bitterleaf	Waterleaf	Cocoyam	Cassava	WHO limits
Pb	0.14 ± 0.00^c^	0.24 ± 0.00^e^	0.22 ± 0.00^d^	0.11 ± 0.00^a^	<0.0001	0.30
Cd	0.01 ± 0.00^a^	0.08 ± 0.00^d^	0.07 ± 0.00^d^	0.02 ± 0.00^c^	<0.0001	0.10
Cr	0.04 ± 0.00^c^	0.20 ± 0.01^e^	0.11 ± 0.00^d^	0.02 ± 0.00^a^	<0.0001	0.05
Mn	4.07 ± 0.00^e^	3.05 ± 0.01^d^	1.92 ± 0.00^b^	0.67 ± 0.00^a^	0.89 ± 0.01^c^	–
Fe	256 ± 0.26^e^	0.82 ± 0.00^a^	1.35 ± 0.00^c^	10.5 ± 0.01^d^	<0.0001	–
Zn	1.22 ± 0.01^c^	0.21 ± 0.01^a^	0.13 ± 0.00^d^	1.58 ± 0.06^e^	0.10 ± 0.00^b^	100

**Table 8:** Mean levels (mgkg^−1^) of studied metals in test area (Gokana)

	Soil	Bitterleaf	Waterleaf	Cocoyam	Cassava	WHO limits
Pb	0.39 ± 0.00^d^	0.48 ± 0.05^e^	0.29 ± 0.00^a^	0.37 ± 0.04^c^	0.20 ± 0.00^b^	0.30
Cd	0.02 ± 0.00^b^	0.04 ± 0.00^d^	0.03 ± 0.00^a^	0.03 ± 0.00^a^	0.02 ± 0.00^c^	0.10
Cr	0.01 ± 0.00^a^	0.07 ± 0.00^b^	0.04 ± 0.00^d^	<0.0001	<0.0001	0.05
Mn	0.78 ± 0.01^a^	1.18 ± 0.03^b^	1.19 ± 0.02^b^	2.73 ± 0.02^c^	0.37 ± 0.00^a^	–
Fe	284 ± 0.60^e^	6.54 ± 0.02^b^	4.74 ± 0.04^a^	23.6 ± 0.07^d^	16.0 ± 0.03^c^	–
Zn	0.90 ± 0.00^a^	2.32 ± 0.00^b^	1.81 ± 0.00^c^	2.94 ± 0.00^d^	0.98 ± 0.00^e^	100

## Conclusions

Potentially carcinogenic hydrocarbons and heavy metals pollution in Gokana could be attributed to anthropogenic heavy metals enrichment of the oil-rich industrialized state, as well as oil exploration and related ill practices sighted during the study. Humans and grazing animals may be in serious danger due to exposure along the food chain, and urgent need for remediation strategies and management of these contaminated zones is implied. In this study, 24 plant samples, besides soils, were collected from environmentally disturbed and undisturbed communities and analysed for PAHs, TPH and some metals. The results of the statistical analyses show that with respect to PAHs, the eight datasets are significantly different (*P* ≤ 0.05) and can be considered dissimilar dataset representative of disturbed and undisturbed environments. Farmers can suffer direct exposure from such contaminated soils besides ingestion of food items, which is boundless, and immediate action, therefore, is needed following the results obtained from secondary evaluation of carcinogenic hydrocarbons distribution in study soils.
